# Silicon-based planar devices for narrow-band near-infrared photodetection using Tamm plasmons

**DOI:** 10.1515/nanoph-2024-0062

**Published:** 2024-04-18

**Authors:** Wenyue Liang, Yajin Dong, Long Wen, Yongbing Long

**Affiliations:** Institute of Nanophotonics, 47885 Jinan University, Guangzhou, 511443, China; College of Electronic Engineering, 12526 South China Agricultural University, Guangzhou, 510642, China

**Keywords:** near-infrared photodetector, silicon photonics, Tamm plasmon, hot electron, Schottky junction

## Abstract

Designing efficient narrow-band near-infrared photodetectors integrated on silicon for telecommunications remains a significant challenge in silicon photonics. This paper proposes a novel silicon-based hot-electron photodetector employing Tamm plasmons (Si-based TP-HE PD) for narrow-band near-infrared photodetection. The device combines a one-dimensional photonic crystal (1DPC) structure, an Au layer, and a silicon substrate with a back electrode. Simulation results show that the absorption of the TP device with a back electrode is 1.5 times higher than without a back electrode, due to increased absorption from multiple reflections between the back electrode and the 1DPC structure. Experimentally, the responsivity of the fabricated device reaches 0.195 mA/W at a wavelength of 1400 nm. A phenomenological model was developed to analyze the photoelectric conversion mechanism, revealing reasonable agreement between the theoretically calculated and experimentally measured internal quantum efficiencies. Additional experiments and simulations demonstrate the tunability of the resonance wavelength from 1200 nm to 1700 nm by adjusting structural parameters. The Si-based TP-HE PD shows potential for silicon-based optoelectronic applications, offering the advantages of a simple structure, low cost, and compatibility with silicon photonic integrated circuits. This work represents the first demonstration of a silicon-based hot electron NIR photodetector utilizing Tamm plasmons.

## 1 Introduction

Narrow-band near-infrared (NIR) photodetectors have a wide range of applications in various fields, such as optical telecommunications, medical diagnostics, light detection and ranging, and sensors, due to their ability to detect light in a specific wavelength range with high sensitivity and accuracy [1], [2], [3], [4], [5], [6]. The integration of silicon photonics with electronic functionalities is a promising solution to overcome the limitations of electrical interconnects in modern integrated circuits. However, designing efficient photodetectors integrated on silicon for telecommunications remains a significant challenge. Developing silicon-based narrow-band near-infrared (NIR) photodetectors is crucial for silicon-based optoelectronic integration and future on-chip optoelectronic devices, as they enable optical telecommunications, medical diagnostics, light detection and ranging, and sensors in the specific wavelength range with high sensitivity and accuracy [1], [2], [3], [4], [5], [6].

Organic photodetectors (OPDs) with narrow-band NIR detection have been reported through the strategies of charge collection narrowing, exciton dissociation narrowing, charge injection narrowing, use of an optical microcavity, use of narrow-band organic semiconductor materials and transmittance region engineering [7], [8], [9], [10]. These devices show advantages such as low-cost and easy manufacturing, flexibility, easily adjustable spectral response range, and lightweight. In addition, OPDs show the potential for fabrication on Si substrates and are compatible with complementary metal oxide semiconductors (CMOSs) [11], [12]. However, the widespread commercial adoption of OPDs is hindered by limitations in the electrical properties of organic semiconductor materials, stability in air, and reliable integration. However, OPDs are still not widely used commercially due to limitations of the electrical properties of the organic semiconductor material, stability in air, and reliable integration [13], [14].

In contrast, mainstream NIR photodetectors are based on inorganic semiconductor materials, such as germanium, indium gallium arsenic, and indium phosphide, exhibit the advantages of high carrier mobility, low exciton binding energy, and better stability. However, these inorganic photodetectors (IPDs) are unavailable for spectrally selective photodetection directly due to the broad absorption spectrum of the semiconductor materials [15]. Detecting specific wavelengths typically requires additional optical elements like filters, resulting in complex optical structures and higher costs. Moreover, the fabrication process of these devices is facing the issues of incompatible with Si-based photonic integration platforms, making integration with other optoelectronic devices challenging. The complex, difficult, and costly hybrid integration process limits the widespread application of these devices.

The photoelectric conversion mechanism of intraband transition of hot carriers (hot electrons or hot holes) based on surface plasmons excited by metal materials has emerged as a candidate for realizing silicon-based NIR optoelectronic devices [16], [17], [18], [19], [20], [21]. Through the photoelectric conversion mechanism of hot carrier emission in metal–semiconductor or metal–insulator heterojunction structures, the photodetection cutoff wavelength of silicon-based hot carrier photodetectors can be extended to 2 μm [22]. By carefully designing subwavelength nanostructures such as nanogratings [23], [24], [25], nanowires [26], and nanotrenches [1] to excite plasmonic resonances, narrow-band NIR photodetection can be achieved. Among them, metal-silicon Schottky junction devices incorporating plasmonic nanostructures have garnered significant interest for sub-bandgap photodetection in silicon. However, the fabrication processes for these devices are complicated and costly. As an alternative, planar hot carrier photodetectors with the potential advantages of a simple structure and low cost have been proposed [27], [28], [29], [30]. Tamm plasmons (TP) generated by metal/distributed Bragg reflector (DBR) or one-dimensional photonic crystals (1DPCs) multilayer planar structures can trap light and exhibit narrow-band optical resonance absorption and have been used to obtain TP-based hot-electron photodetectors [31], [32], [33], [34], [35], [36], [37], [38], [39], [40]. However, an experimental demonstration of a Si-based hot electron NIR photodetector based on TPs has not been reported.

In this paper, we investigate a narrow-band planar hot-electron photodetector by combining the plasmon mechanism of the planar structure with the rectifying properties of Au/silicon Schottky junctions (Si-based TP-HE PD) both theoretically and experimentally. As a result of the TP excitation in the device, the total absorption can reach over 76.5 % with a full width at half maximum (FWHM) of approximately 52 nm. Correspondingly, the responsivity of the device reaches 0.11 mA/W at 1450 nm. Furthermore, by changing the incident angle, the resonant peak can be tuned to 1400 nm, and a photoresponsivity of 0.195 mA/W is achieved. This work demonstrates the potential of utilizing Tamm plasmons in conjunction with metal-silicon junctions for realizing efficient and tunable silicon-based narrow-band NIR photodetectors.

## 2 Device design and optical analysis

**Figure 1:**
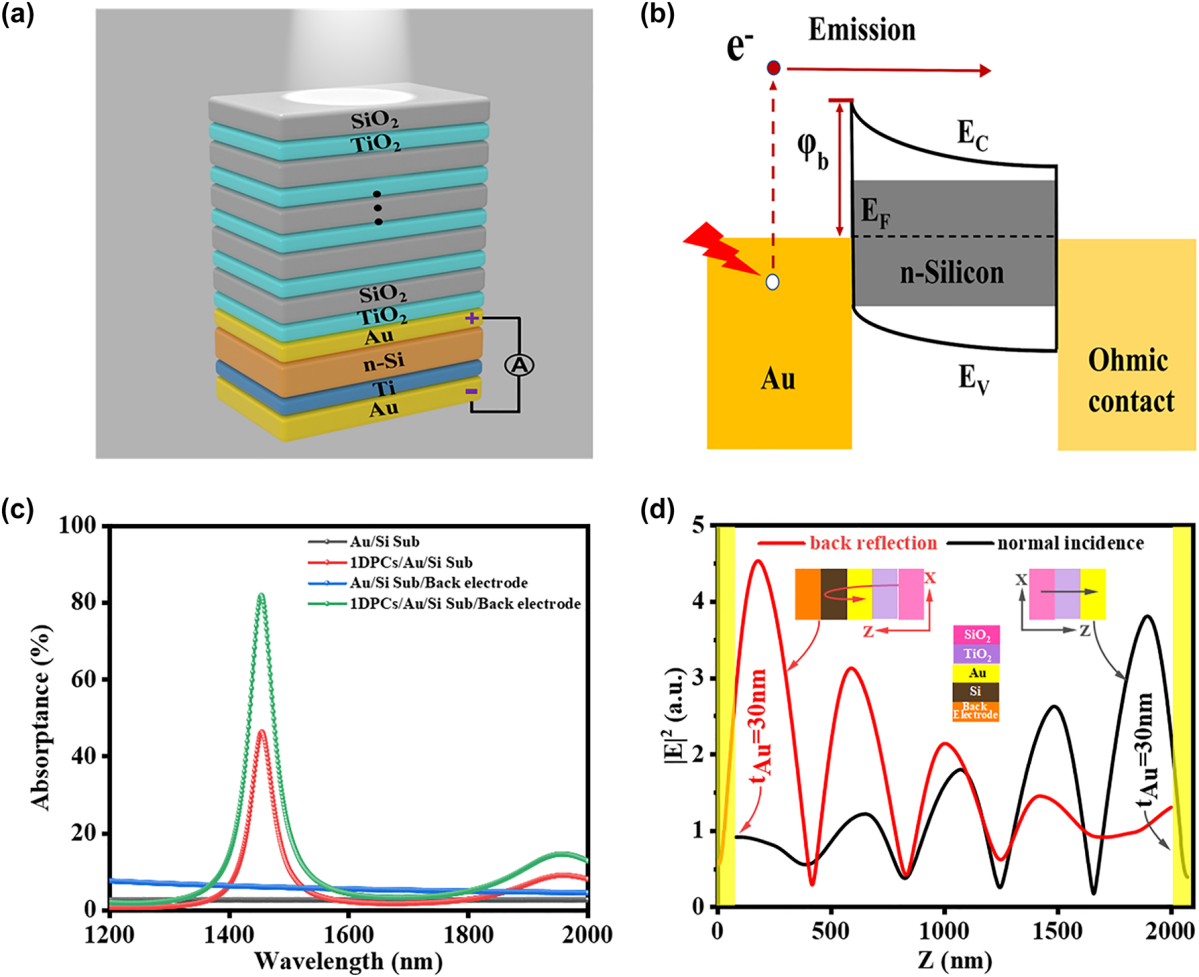
Device structure design and optical properties of the Si-based TP-HE PD. (a) Schematic diagram of the proposed TP-based hot-electron photodetector. The 1DPCs consists of 5 pairs of alternating SiO_2_/TiO_2_ layers, and the theoretically designed SiO_2_ and TiO_2_ layers are 244 nm and 165 nm thick, respectively. (b) Schematic illustration of the band structure of the TP-HE PD. The Schottky barrier is denoted as *φ*_*b*_. (c) Absorption spectra *A*(*λ*) of the Au/Si sub structure, 1DPCs/Au/Si sub structure without the back electrode, and the 1DPCs/Au/Si sub structure with the back electrode. (d) Profile of the normalized electric field ${\left|E\right|}^{2}$ along the *z*-axis for TPs at the interface between a 5-pair 1DPCs and 30 nm thick Au under front-side incidence and back-side reflection incidence, where the back-reflection interface is Si/back electrode.

Figure 1a shows a schematic diagram of the proposed Si-based TP-HE PD, which consists of a 1DPCs structure, an Au thin film, and a silicon substrate with a back electrode. The 1DPCs consists of 5 pairs of alternating SiO_2_/TiO_2_ layers with a central wavelength (*λ*_1DPCs_) of 1382.5 nm, and each layer has a quarter-wavelength optical thickness (*λ*_1DPCs_/4*n*, where *n* is the refractive index). The thickness of the Au interlayer *(d*_Au_) is set as 30 nm, and the back electrode (20 nm Ti/100 nm Au) is in ohmic contact with silicon on the side of silicon. The energy band diagram of the structure is shown in Figure 1b. Upon absorption of infrared light photon energy, hot electrons are generated in the thin Au layer, and those with sufficient energy to overcome the Schottky barrier (*φ*_*b*_) can be ejected into the silicon, contributing to the photocurrent. Notably, hot electron generation is mainly contributed by the absorption in the thin Au layer under normal incidence and multiple back-reflection incidence. First, the simulation absorption spectra *A* of the Au/Si Sub structure, 1DPCs/Au/Si Sub structure without the back electrode, and the 1DPCs/Au/Si Sub structure with the back electrode are shown in Figure 1c as a function of wavelength (*λ*). The back electrode serves as both an electrode and a mirror to enhance device absorption. When it is just an Au/Si structure, the absorption is less than 3 % due to the absence of optical resonance. Even using the back electrode as a reflector, the absorption is still below 10 %. When incorporating 1DPCs into the Au/Si structure, the absorption increased to more than 45 % at the resonant peak of 1454 nm, attributed to the excitation of Tamm plasmon. Further utilizing the back electrode as a reflector, the absorption increases to about 80 % and the full width at half maximum (FWHM) of the TP resonance is approximately 52 nm. To further clarify the field distribution of TP resonance, the vertical profile of the normalized electric field ${\left|E\right|}^{2}$ along the *z*-axis for the TP resonance at the interface between a 5-pair 1DPCs and a 30 nm Au film under normal incidence and multiple back-reflection incidence is shown in Figure 1d. Under front-side incidence and back-side reflection incidence both excite Tamm plasmon with a strongest electric field occurs near to the Au/Si interface and rapidly decays away from it.

**Figure 2:**
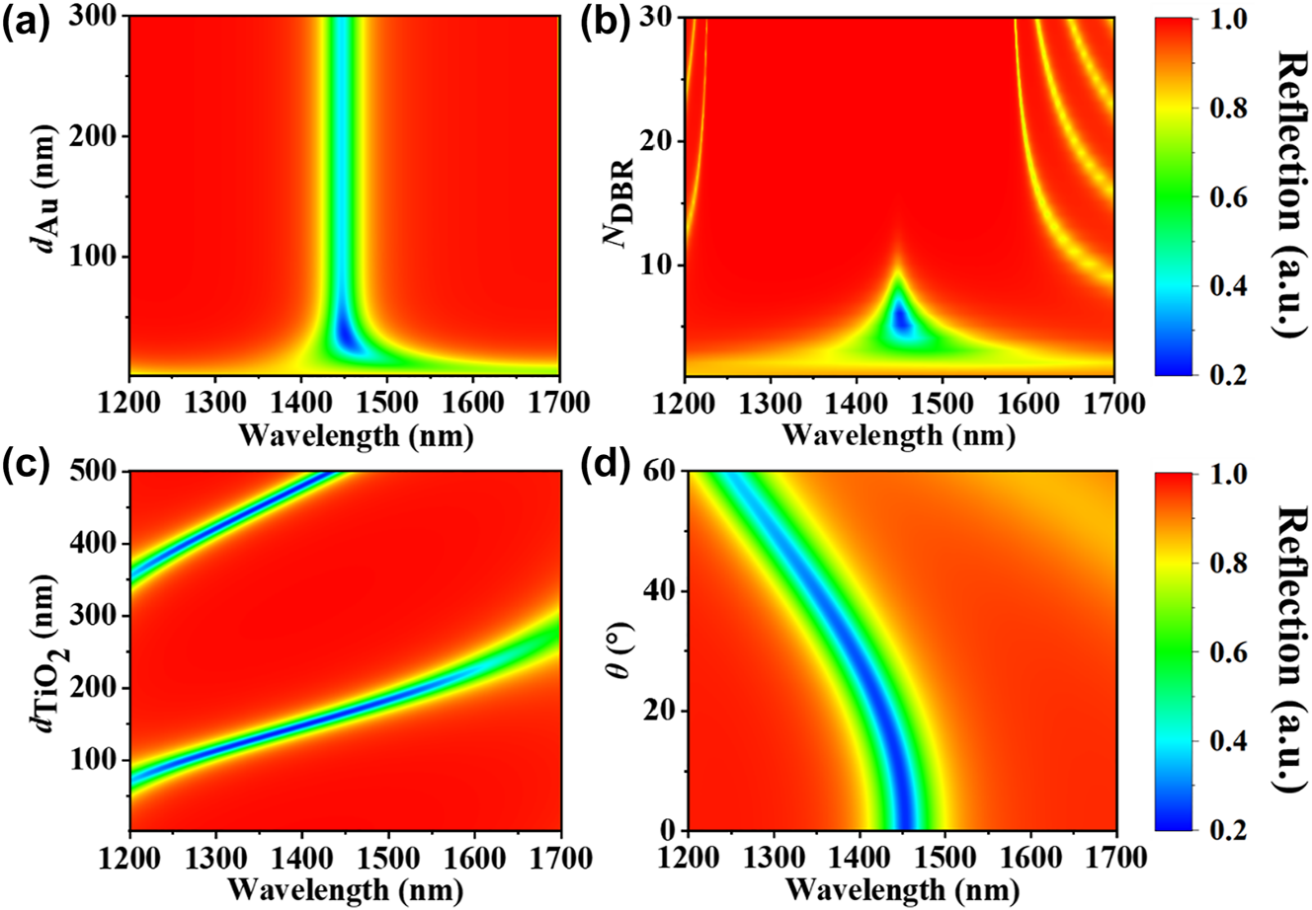
Dependences of the TP device reflection *R*(*λ*) on (a) the Au film thickness (*d*_Au_), (b) the number of pairs of the 1DPCs (*N*_1DPCs_), (c) the TiO_2_ film thickness (*d*_TiO2_), and (d) the incident angle (*θ*).

After confirming the excitation of strong TPs by the 1DPCs/Au structure, the effect of different parameters on the resonance must be investigated to determine, for example, how to adjust the thickness of Au (*d*_Au_) and the number of pairs of the 1DPCs (*N*_1DPCs_) to achieve stronger TP field localization (Figure 2a and b) and how to achieve resonance peak tuning through the TiO_2_ thickness and incident angle (Figure 2c and d). Figure 2a exhibits the dependence of the TP device reflection *R*(*λ*) on the Au film thickness *d*_Au_. The greater the thickness of Au is, the worse the contrast of its reflection spectra. The contrast of the reflectance spectrum is the greatest when the Au thickness is in the range of 20–50 nm. In the experiment, the thickness of the Au film was chosen to be 30 nm due to the influence of hot electron transport loss. Moreover, *N*_1DPCs_ has a great influence on the reflection performance of the device, as shown in Figure 2b. Only numbers of 1DPCs pairs in the range from 3 to 7 have significant excitation of TP. However, with a large *N*_1DPCs_, most of the incident light will be directly reflected, forming an optical forbidden band. Therefore, a proper *N*_1DPCs_ is crucial for the realization of strong TP, and 5 pairs of the 1DPCs structure were selected experimentally.

Additionally, the tunability of the TP resonance can be realized by adjusting *d*_TiO2_ and the incident angle *θ*. Figure 2c clearly shows that the TP resonance peak can be modulated from 1200 nm to 1700 nm by adjusting the TiO_2_ thickness, and *λ*_TP_ shows a certain linear dependence on *d*_TiO2_. The separation between two adjacent TP resonances satisfies *λ*/2*n*, where *n* is the refractive index of TiO_2_. Similarly, Figure 2d displays the angular-dependent optical reflection. Strong optical reflection of the TP structure can be sustained over a broad range of incident angles (from 0° to 60°), and the resonance undergoes a blueshift in the 200 nm range with increasing incident angle. Therefore, the device demonstrates excellent angular performance, which is difficult to achieve with conventional planar structures.

## 3 Device fabrication and photoelectric properties

In the experiment, the 1DPCs structure was fabricated on a fused double-polished n-type Si substrate using the following steps. First, an Ohmic contact electrode (20 nm Ti/100 nm Au) was deposited on the side of silicon using DC-magnetron sputtering, with an area of 5 × 5 mm^2^. Second, a gold film of 30 nm was deposited on the other side of the cleaned n-type Si substrate by electron-beam evaporation, with the entire process involving the deposition of thin films on flat structures through a physical shadow mask on top of the structure, with an area of 7 × 7 mm^2^. Finally, 5 pairs of SiO_2_/TiO_2_ layers were alternately deposited on the Au film surface as the active area by electron-beam evaporation. Here, the thicknesses of the SiO_2_ and TiO_2_ films are 244 nm and 165 nm, respectively. Notably, the active area is 5 × 5 mm^2^, which is intended to form the Au film portion as a front electrode.

**Figure 3:**
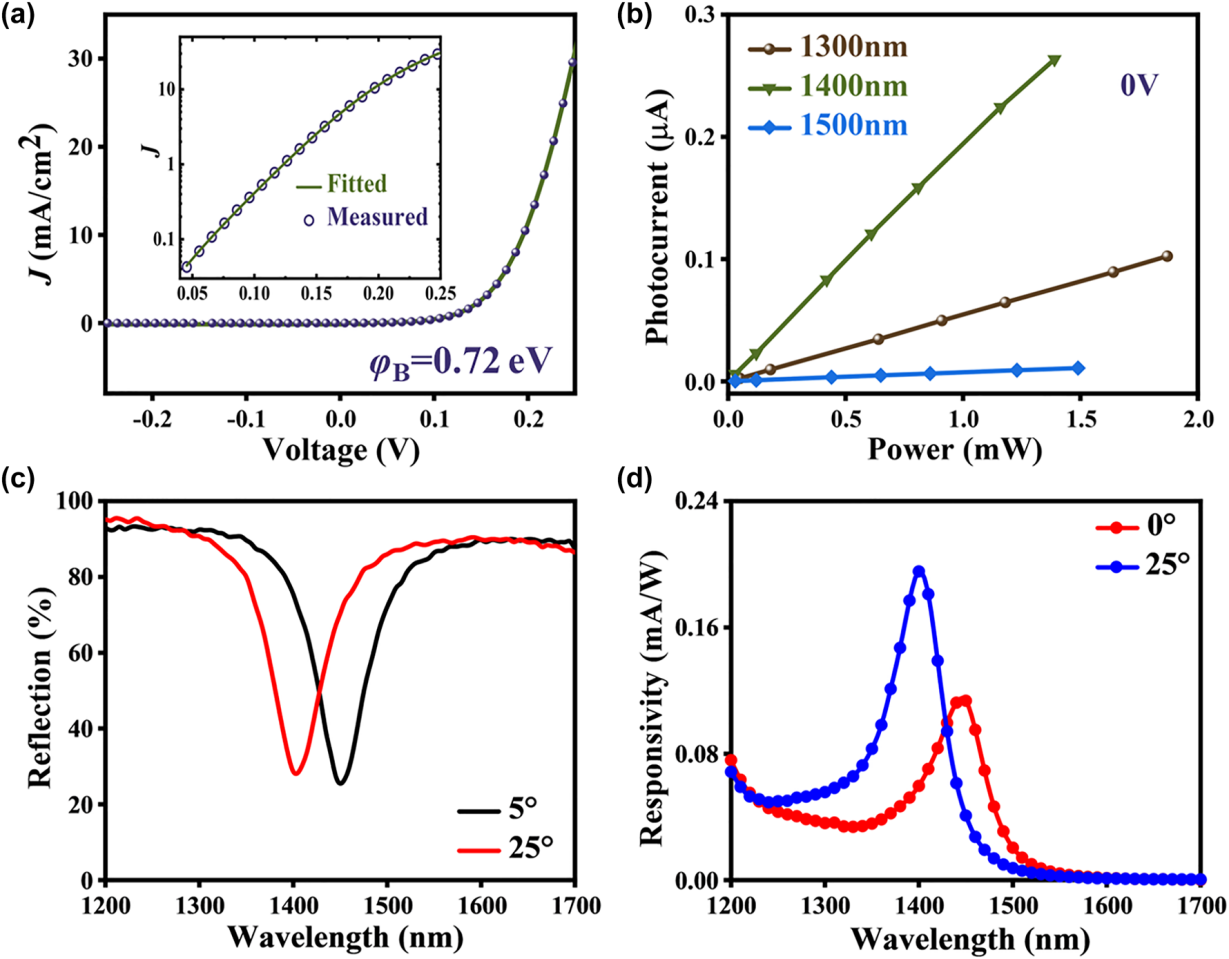
Electrical and optical characterization of the fabricated Si-based TP-HE PD. (a) Measured dark *I*–*V* curves for the proposed TP devices; the inset shows the logarithmic plots. The theoretical fitting data are presented as solid circles. (b) Photocurrent of the TP photodetector as a function of the illumination intensity measured under a bias of 0 V and different illumination wavelengths. (c) Measured reflection spectra of the TP structure under different incident angles (*θ* = 5° and 25°). (d) Measured responsivity under different incident angles (*θ* = 0° and 25°) of devices.

Without illumination, the TP device exhibits clear rectifying behavior in the *I*–*V* plots, as shown in Figure 3a, indicating the formation of a Schottky contact between Au and n-type Si. The dark current of the TP device at −0.2 V is only level of nA. The *I*–*V* curve fitting procedure based on the Richardson–Schottky equation was adapted to quantitatively extract the barrier height of the Schottky barrier devices, and the fitted *I*–*V* curve is shown in the inset of Figure 3a. For a Schottky barrier diode with the assumption that the current is due to thermionic emission, the relation between the applied forward bias and current can be expressed as:

(1) $$I=I_{s}\left[\exp\left(\frac{q\left(V-IR_{s}\right)}{nkT}\right)-1\right]$$

(2) $$I_{s}=SA^{*}T^{2}\exp\left(\frac{-q\varphi_{B}}{kT}\right)$$

where *S* is the active area of the measured device (5 × 5 mm^2^), A* is the Richardson constant (120 A/cm^2^ K [2] for silicon), *T* is the temperature (300 K), *φ*_*B*_ is the barrier height, *k* is the Boltzmann constant, *n* is the ideality factor, and *R*_*S*_ is the device resistance (contact resistance and series resistance of bulk silicon). The key electrical parameters of the device can be extracted by fitting the measured dark *I*–*V* curve of the TP devices to the above thermionic emission equations. The derived effective barrier height of the TP device is 0.72 eV. During the testing process, the output lasing wavelength of the supercontinuum laser (NKT Photonics SC-400) was adjusted with an acousto-optic tunable filter (AOTF) in the range of 1200–1700 nm, and the photocurrent was sampled in real-time with a Keithley 2636B SourceMeter. The illumination power in the device’s active region at a single wavelength was at the milliwatt level. All the measurements were conducted at zero bias, and the recorded photocurrents showed a quick photoelectric response. To quantitatively compare the contributions of the plasmonic resonance and background responses, we examine the photocurrent at the resonance wavelength relative to a non-resonance wavelength, as shown in Figure 3b. The photocurrent of the TP photodetector as a function of the illumination intensity measured at different illumination wavelengths and shows that the photocurrent is not linearly related to the illumination wavelength when the incident light power is constant. For example, at 1 mW incident power, the photocurrent is 0.195 μA at the resonance wavelength of 1400 nm, which is over 3.5 times higher than the 0.055 μA photocurrent at the non-resonance wavelength of 1300 nm. This demonstrates that the resonant plasmonic hot electron emission is the dominant mechanism responsible for the photocurrent enhancement at the resonance wavelength, while the background intraband absorption has a much smaller contribution. The figure also shows the photocurrent linearly varies with the illumination power, which demonstrates that there is an efficient electron transport path between the Au and Si Schottky contact.

Figure 3c shows the measured reflection spectra of the TP structure under different incident angles (*θ* = 5° and 25°). The TP resonance in the TP structure appears to blueshift as the angle increases, which is consistent with the simulation results. In detail, the measured reflection spectra indicate TP resonance at 1450 nm and 1400 nm under incident angles *θ* = 5° and *θ* = 25°, respectively. At *θ* = 5°, the TP device shows a reflection of approximately 25 % at a wavelength of 1450 nm, which is over 4 times lower than the reflection at a wavelength of 1300 nm, which means higher absorption and photocurrent at a wavelength of 1450 nm than at a wavelength of 1300 nm. At *θ* = 25°, the TP resonance wavelength is tuned to 1405 nm, and the reflection is approximately 28 %. The shifts of the resonance peaks under different incident angles in the experiments are consistent with those in the theoretical calculation, as shown in Figure 2d. The measured responsivities at different wavelengths of devices under incident angles *θ* = 0° and *θ* = 25° are shown in Figure 3d, which are in good agreement with the experimentally measured reflection spectrum. The position of the TP resonance in the optical response spectra is consistent with that in the reflection spectra. The measured responsivities of the TP device at *λ* = 1450 nm and *λ* = 1400 nm are 0.11 mA/W and 0.195 mA/W for *θ* = 0° and *θ* = 25°, respectively. The background response at non-resonance wavelengths (such as near the 1200 nm) originates from the absorption of the metal layer itself, specifically from intraband transitions caused by direct absorption of the metal under light excitation. These intraband transitions generate hot carriers with energy close to the Fermi level. For our metal/n-type silicon device, the effective carriers are electrons. However, the probability of these low-energy electrons overcoming the Schottky barrier to be emitted into the semiconductor is relatively low. In contrast, the electrons generated by the metal absorption caused by the plasmonic resonance originate from interband transition. The plasmonic resonance generates hot electrons with much higher energy compared to those from direct intraband absorption. These high-energy hot electrons have a significantly greater probability of being emitted over the Schottky barrier into the semiconductor, resulting in the enhancement of the photocurrent at resonance wavelengths. Furthermore, we fabricated a device (Device 2) with a 1DPC structure designed for a central resonant wavelength of 1580 nm, which is provided in the Supplementary Material (Figure S2). The time-dependent photocurrent of Device 2 was measured at 0 V bias under illumination wavelengths ranging from 1460 nm to 1640 nm (Figure S2a in the Supplementary). The measured responsivity of Device 2 at 0 V and −1 V bias was 0.01 mA/W and 0.012 mA/W, respectively, at the resonance peak of 1580 nm (Figure S2b in the Supplementary).

## 4 Theoretical analysis of the photoelectric conversion mechanism

**Figure 4:**
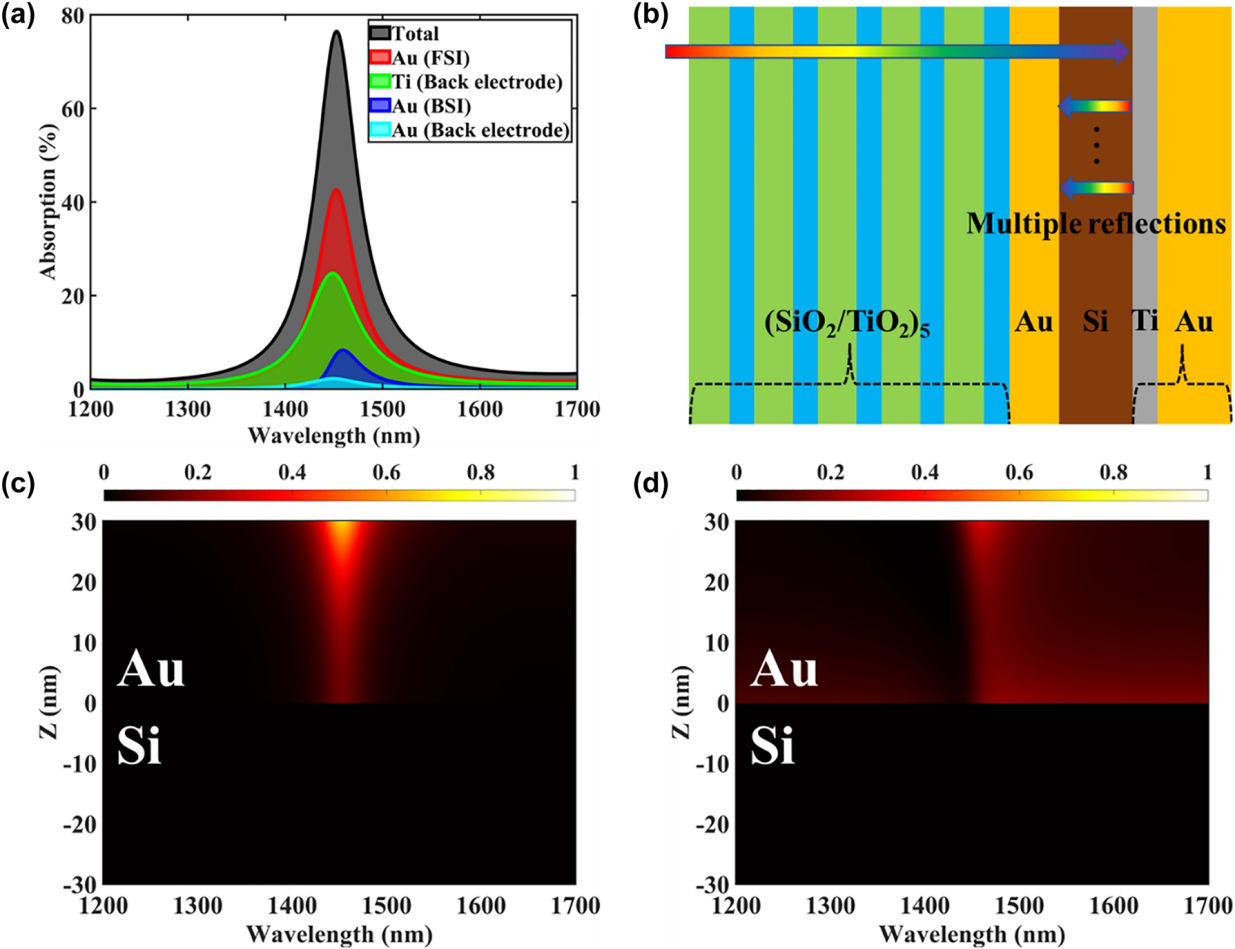
Hot electron generation and absorption mechanism in the Si-based TP-HE PD. (a) Simulated absorption spectra for the fabricated structure showing the fractions of light absorbed by different absorbing materials in the device, including the BSI contribution of the 30 nm Au layer. (b) Schematic diagram of multiple reflections of incident light inside the device. Normalized hot electron generation rate distribution for incident light under (c) FSI and (d) BSI.

To understand the photoelectric conversion mechanism, we calculated the absorption spectra within the device, and the results are shown in Figure 4a. The total absorption of the device reaches a maximum of 76.5 % at the resonance wavelength of 1453 nm, which is contributed by four parts: 30 nm Au layer absorption under front-side incidence (FSI) and back-side reflection incidence (BSI) with multiple reflections and absorption in the Ti layer and Au layer in the back electrode. The absorption in the 30 nm Au layer under FSI reaches a value of 42.6 % at the resonance wavelength, at which the TPs are excited. To further improve the Au layer absorption, the back electrode acts as a mirror to realize the BSI of the transmitted light with multiple reflections, as shown in Figure 4b. Since BSI occurs in the Si substrate, the absorption peak is slightly shifted to 1459 nm, and the absorption in the 30 nm Au layer is increased by 8.3 %. In addition, due to the effect of multiple reflections, the transmitted light is also incident multiple times on the back electrode composed of Ti/Au, and there is more than 25 % absorption at the resonance wavelength, where the absorption is mainly in the Ti layer. The metal layer in the device will generate hot electrons after absorbing photons. In this case, the hot electrons generated in the 30 nm Au layer are mainly analyzed, and the light absorption in the back electrode does not contribute to the photoelectric conversion of the device. The generation rate for hot electrons $G\left(z,v\right)$ can be calculated as [29]:

(3) $$G\left(z,v\right)=\frac{\varepsilon_{i}\left|E\left(z,v\right)\right|}{h/\pi }$$

where *v* is the frequency of the photon, *ε*_*i*_ is the imaginary part of the dielectric constant of Au, *E*(*z*,*v*) is the electric field at position *z* and frequency *v*, and *h* is the Planck constant.

Figure 4c shows the generation rate distribution of hot electrons in the 30 nm Au layer at different wavelengths under FSI. The generation rates are normalized to the total hot electron generation rate distribution including the generation rates under FSI (*G*_*f*_) and BSI (*G*_*b*_). Hot electrons are mainly generated at the resonance wavelength, most of them are distributed near the Au/TiO_2_ interface, and the distribution along the negative direction of *Z* presents an exponential decay. Equation (3) shows that this distribution is determined by the internal electric field, as shown in Figure 1c. The hot electron generation rate distribution under BSI is somewhat different from that under FSI, as shown in Figure 4d. Hot electrons are mainly generated near the Au/TiO_2_ interface at the TP resonance wavelength. However, for a specific position inside the Au layer, the distribution of hot electron generation rates is similar to the asymmetric Fano line shape around the resonance wavelength. In the direction from the resonance wavelength toward long wavelengths, hot electrons are generated close to the Au/Si interface. This is due to the coupling between the excitation of TPs under BSI and the cavity formed by the 1DPCs and the back electrode. In addition, according to *G*_*f*_ and *G*_*b*_, we can calculate the total number of hot electrons (*N*_*g*_) generated in the 30 nm Au layer.

**Figure 5:**
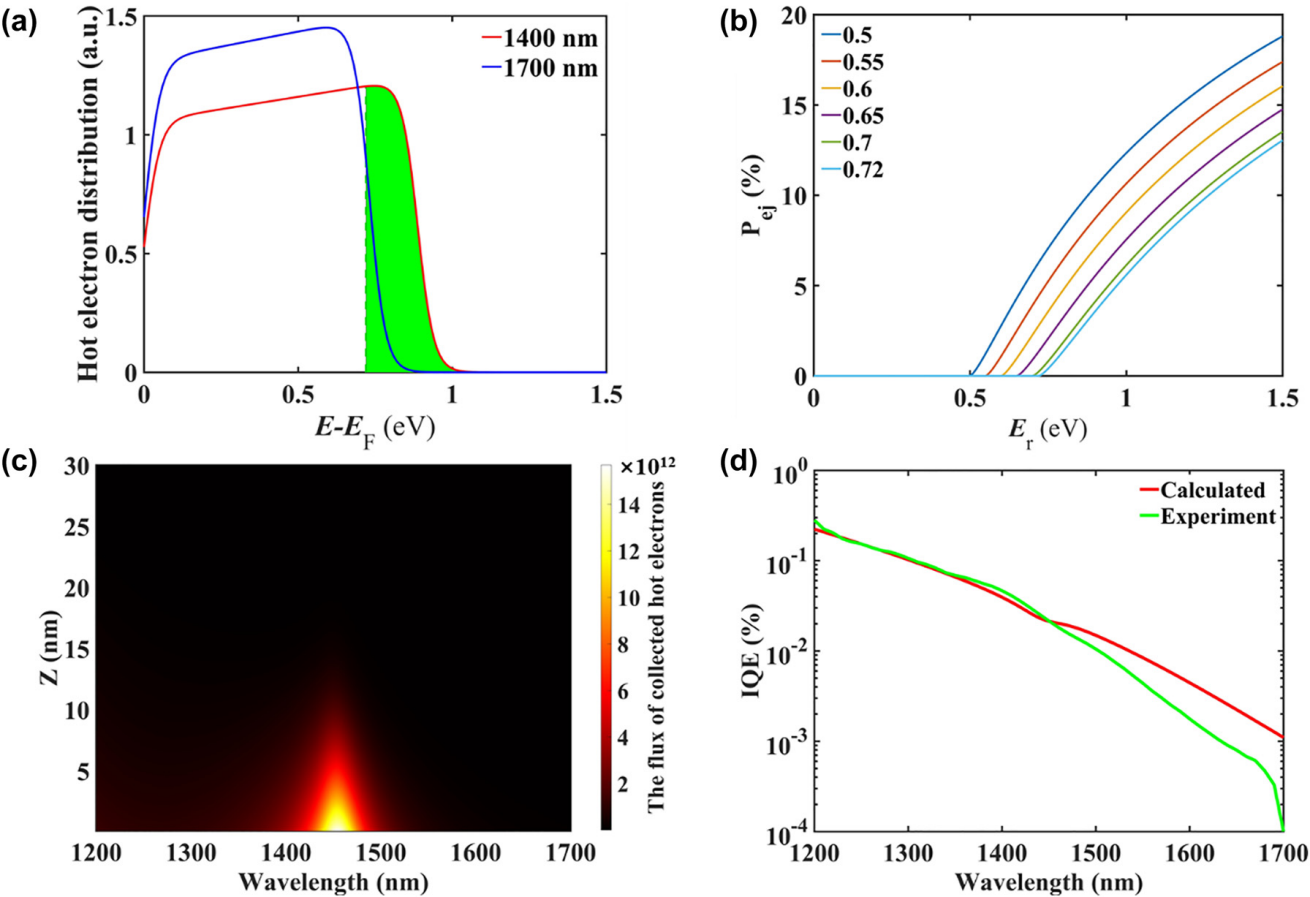
Theoretical analysis of the Si-based TP-HE PD by the phenomenlogical model. (a) Initial energy distribution of hot electrons with wavelengths of 1400 nm and 1700 nm. (b) Impact of the barrier height (varying from 0.5 eV to 0.72 eV) on the ejection probability of hot electrons with different energies reaching the Au/Si interface. (c) The flux of collected hot electrons at different positions in the Au layer at different wavelengths. (d) IQEs versus illumination wavelength of the TP devices predicted by the phenomenological model.

The energy distribution of the hot electrons is critical because only hot electrons with sufficient energy and momentum can cross the Au/Si Schottky barrier and be collected. The hot electron initial energy density distribution upon photon excitation can be determined by the function of the electron distribution joint density of states (EDJDOS) [41], [42]:

(4) $$P_{d}\left(E\right)\propto D\left(E-hv\right)f\left(E-hv\right)D\left(E\right)\left(1-f\left(E\right)\right)$$

(5) $$f\left(E\right)=\frac{1}{1+e^{E-\frac{E_{f}}{kT}}}$$

where *E* is the energy of the excited electron, *D*(*E*) is the electron density of states depending on the electron energy, *hv* is the energy of the incident photon, *f*(*E*) is the Fermi distribution function, and *E*_*f*_ is the Fermi level (5.53 eV for Au). The calculated EDJDOS upon illumination at wavelengths of 1400 nm and 1700 nm for Au are plotted in Figure 5a. The green area indicates hot electrons with energy higher than the Schottky barrier. Compared with the wavelength of 1700 nm, more hot electrons with higher initial energy are generated at the wavelength of 1400 nm, and these hot electrons have a higher probability of emission. After hot electrons are generated in the Au layer, half of the hot electrons move toward the Au/Si interface. The Schottky barrier at the Au/n-Si contact significantly deviates from the predictions of the Mott–Schottky theory due to the presence of a high density of surface states at the Au–Si interface. Therefore, the effect of different barrier heights on the probability of hot electron emission must be considered. The ejection probability *P*_*ej*_ of hot electrons at the M–S junction depending explicitly on their excitation energy and Schottky barrier height can be calculated according to the method presented in refs. [43], [44]. The impact of the barrier height on the ejection probability of hot electrons with different energies reaching the Au/Si interface is shown in Figure 5b. According to the graph, with the decrease in Φ_*B*_ from 0.72 eV to 0.5 eV, *P*_*ej*_ continuously grows, as more excited states can gain sufficient energy to overcome the barrier, which is consistent with the theoretically expected results.

(6) $$N_{\text{col}}\left(Z\right)=\overset{hv}{\underset{\varphi_{B}}{\int }}P_{d}\left(E\right)\times N_{g}\left(E,Z\right)\times P_{ej}\left(E,Z\right)\text{d}E$$

Figure 5c shows the flux of total collected hot electrons (*N*_col_) at different positions in the Au layer at different wavelengths calculated from Eq. (6). The collected hot electrons are mainly distributed in the Au layer within a thickness of 10 nm near the resonance peak position (approximately 1400–1500 nm). Finally, the internal quantum efficiency (IQE) can be evaluated by considering all events, including hot carrier generation, transport, and emission.

(7) $$\eta_{\text{IQE}}=\frac{\overset{d_{\text{Au}}}{\underset{0}{\int }}N_{\text{col}}\left(Z\right)\text{d}Z}{\overset{d_{\text{Au}}}{\underset{0}{\int }}\overset{hv}{\underset{0}{\int }}N_{g}\left(E,Z\right)\times P_{d}\left(E\right)\text{d}E\text{d}Z}$$

The results calculated from Eq. (7) and the measured IQE of the TP devices are plotted in Figure 5d. The theoretical fittings reasonably agree with the experimental results.

## 5 Conclusions

In summary, we have proposed and demonstrated a novel approach for hot-electron photodetection in the near-infrared region by integrating a one-dimensional photonic crystal (1DPC), an Au layer, and a Silicon substrate with a Ti/Au back-reflecting electrode. The Si-based TP-HE PD is investigated through theoretical simulation and experimental validation. By designing appropriate structural parameters of the 1DPC, the resonance peak can be tailored to a specific wavelength and tuned by varying the incident angle. At the resonance wavelength of 1400 nm, a responsivity of 0.195 mA/W was achieved at a bias of 0 V. To elucidate the photoelectric conversion mechanism, a phenomenological model was developed, showing good agreement with the experimental results.

Further experiments and simulations demonstrate the tunability of the resonance wavelength from 1200 nm to 1700 nm by adjusting structural parameters. The measured responsivity of a device with a central resonant wavelength of 1580 nm reached 0.01 mA/W at 0 V bias and 0.012 mA/W at −1 V bias. These findings demonstrate the feasibility and application potential of the Si-based TP-HE PD in the NIR band. It showcases compatibility with silicon substrates, a simpler device structure, wavelength tunability, and efficient hot-electron collection.

In conclusion, this work represents a silicon-based hot electron NIR photodetector utilizing Tamm plasmons. The Si-based TP-HE PD shows great potential for silicon-based optoelectronic applications, offering advantages of a simple structure, low cost, and compatibility with silicon photonic integrated circuits. These findings demonstrate the feasibility and application potential of the Si-based TP-HE PD in the NIR band, for the development of efficient and tunable silicon-based narrow-band NIR photodetectors.

## Supplementary Material

Experiments (device fabrication, electrical characterization of Device 2); The simulated reflectance spectra and responsivities of devices with different resonant peak responses; Performance comparison on different TP-based HE NIR photodetectors.

## Supplementary Material

Supplementary Material Details
