# Supplementary material for: Silicon-based planar devices for narrow-band near-infrared photodetection using Tamm plasmons
Source: Nanophotonics. 2024 Apr 18;13(16):2961–70. doi: 10.1515/nanoph-2024-0062 (PMC11502038; doi:10.1515/nanoph-2024-0062)
Supplement: Supplementary file 1 — Supplementary Material Details [file j_nanoph-2024-0062_suppl_001.docx]

Supplementary Material

Silicon-Based Planar Devices for Narrow-Band Near-Infrared Photodetection Using Tamm Plasmons

Wenyue Liang^1,2,#^, Yajin Dong^1,#^ , Long Wen^1,^*, Yongbing Long^2,^*

**^1^**Institute of Nanophotonics, Jinan University, Guangzhou, 511443, China

**^2^**College of Electronic Engineering, South China Agricultural University, Guangzhou, 510642, China

^#^equal contribution

*yongbinglong@126.com, [longwen@jnu.edu.cn](mailto:longwen@jnu.edu.cn)

**S1. Device fabrication**

The fabrication procedure of the Si-based TP-HE PD devices is schematically shown in Figure S1(a). N-type Si wafers <100> were used as the substrates (1-10 Ω·cm, double polished). Prior to Ti/Au deposition, the Si chips were ultrasonically cleaned sequentially in acetone, ethanol, and DI water for 15-20 min. The native oxide, i.e. SiO_2_, was removed from the Si substrates by dilute HF solution (H_2_O: HF = 20:1). After cleanout, the wafers were immediately loaded into the sputtering system in high purity Ar ambient at room temperature, and the cathode contains 20 nm Ti/100 nm Au film were deposited using a shadow mask with an area of ${5\times5 mm}^{2}$. Then, a gold film of 30 nm was deposited on the back side of the silicon substrate by electron-beam evaporation, with the entire process involving the deposition of thin films on flat structures through a physical shadow mask on top of the structure, with an area of $7\times7 \mathrm{mm}^{2}$. Subsequently, the 1DPCs consisting of 5 pairs of SiO_2_/TiO_2_ layers were alternately deposited on the Au film surface as the active area by electron-beam evaporation. Here, the thicknesses of the SiO_2_ and TiO_2_ films are 244 nm and 165 nm, respectively. Notably, the active area is $5\times5 {mm}^{2}$, which is intended to form the Au film portion as a front electrode. All devices were fabricated in the same batch to reduce the influence of fabrication processes.

**
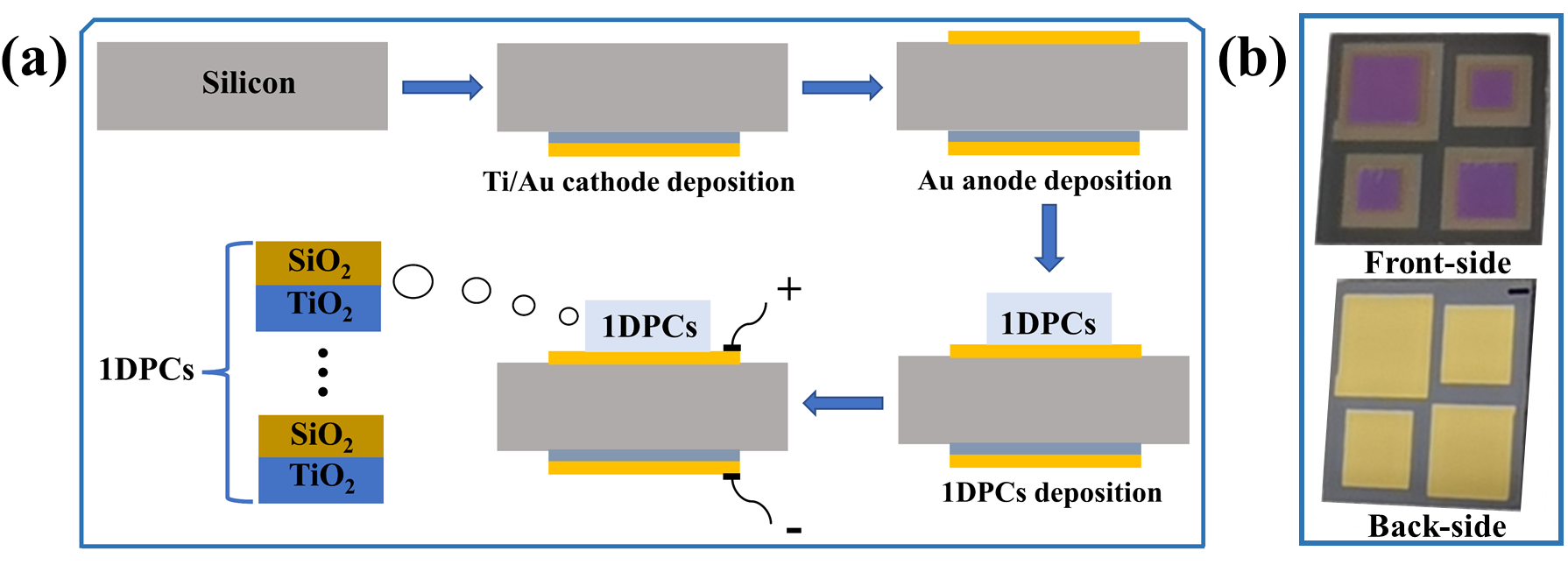
**

**Supplementary Figure S1.** Fabrication of the photodetector. (a) Fabrication procedure for the proposed Si-based TP-HE PD. (b) Pictures of the front-side and back-side of the fabricated device.

**S2. Measured the time-dependent currents and responsivity of the Device 2**

Figure S2 (a) shows the time-dependent currents of device 2 at 0 V in the wavelength range 1460-1640 nm, the 1DPCs consist of 5 pairs of alternating SiO_2_/TiO_2_ layers with a central resonant wavelength (λ_1DPCs_) of 1580 nm. It is found that the maximum value of the photocurrent is 0.1 μA at an incident light of 1580 nm. The measured responsivity under different bias voltage (U=0 V and -1V) of device 2 as shown in Figure S2 (b), the measured responsivity curves show a similar trend as Figure S2 (a). The measured responsivity of device 2 at the position of the resonance peak (*λ* = 1580 nm) at 0 V and -1V bias are 0.01 and 0.012 mA/W, respectively.


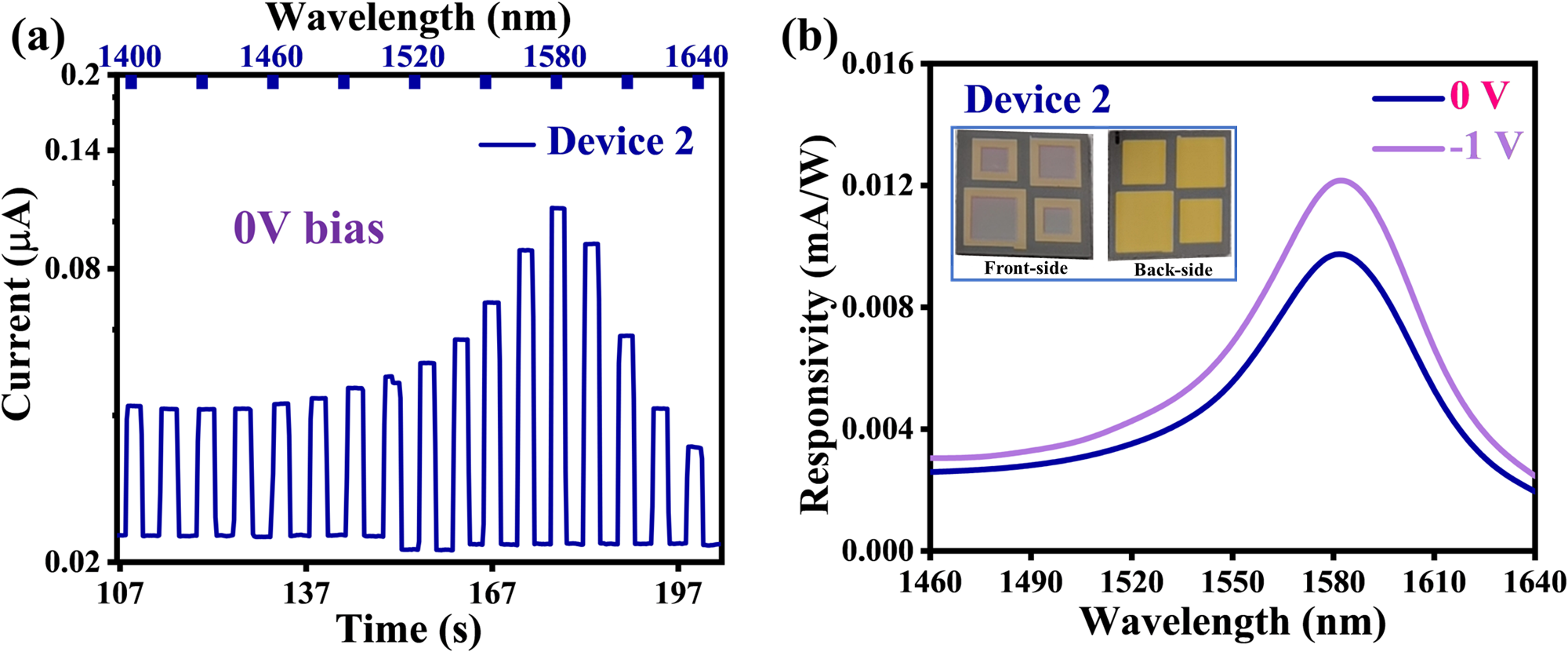


**Supplementary Figure S2.** (a) Time-dependent currents at various illumination wavelengths of device 2 at 0 V, the 1DPCs consist of 5 pairs of alternating SiO_2_/TiO_2_ layers with a central wavelength (λ_1DPCs_) of 1580 nm. (b) Measured responsivity under different bias voltage (U=0V and -1V) of the device 2. The inset shows the front-side and back-side views of the fabricated device.

**S3. The simulated reflectance spectra and responsivities of devices with different resonant peak responses**

To further investigate the device's performance over a broader wavelength range, we designed six devices with different resonant peak responses. The simulated reflectance spectra and responsivities calculated according to Equation 3-7 in the manuscript for these devices are presented in the Figure S3(a) and Figure S3(b), respectively. The reflectance spectra demonstrate that by carefully designing the device structure, we can achieve resonant peaks at various wavelengths spanning from 1200 nm to 1700 nm. Notably, the resonant peaks exhibit narrow bandwidths. This narrowband response is a key feature of our device, enabling wavelength-selective photodetection in the near-infrared region. The responsivities at the resonant peaks range from around 0.75 mA/W to 0.6 μA/W. The height of the Schottky barrier at the Au/Si interface is 0.72 eV, which corresponds to a maximum cut-off wavelength of approximately 1720 nm for the device design. Consequently, the responsivity of the device with a resonance wavelength of 1700 nm is relatively low. To further extend the operating wavelength of the device, alternative metal materials can be employed. For instance, using copper instead of gold can potentially extend the device's operating wavelength up to 2000 nm, as copper forms a lower Schottky barrier with silicon (0.59 eV) we previously reported.^22^ The responsivities at the resonant peaks range from around 7.4 mA/W to 1.9 mA/W when the height of the Schottky barrier decrease to 0.5 eV.


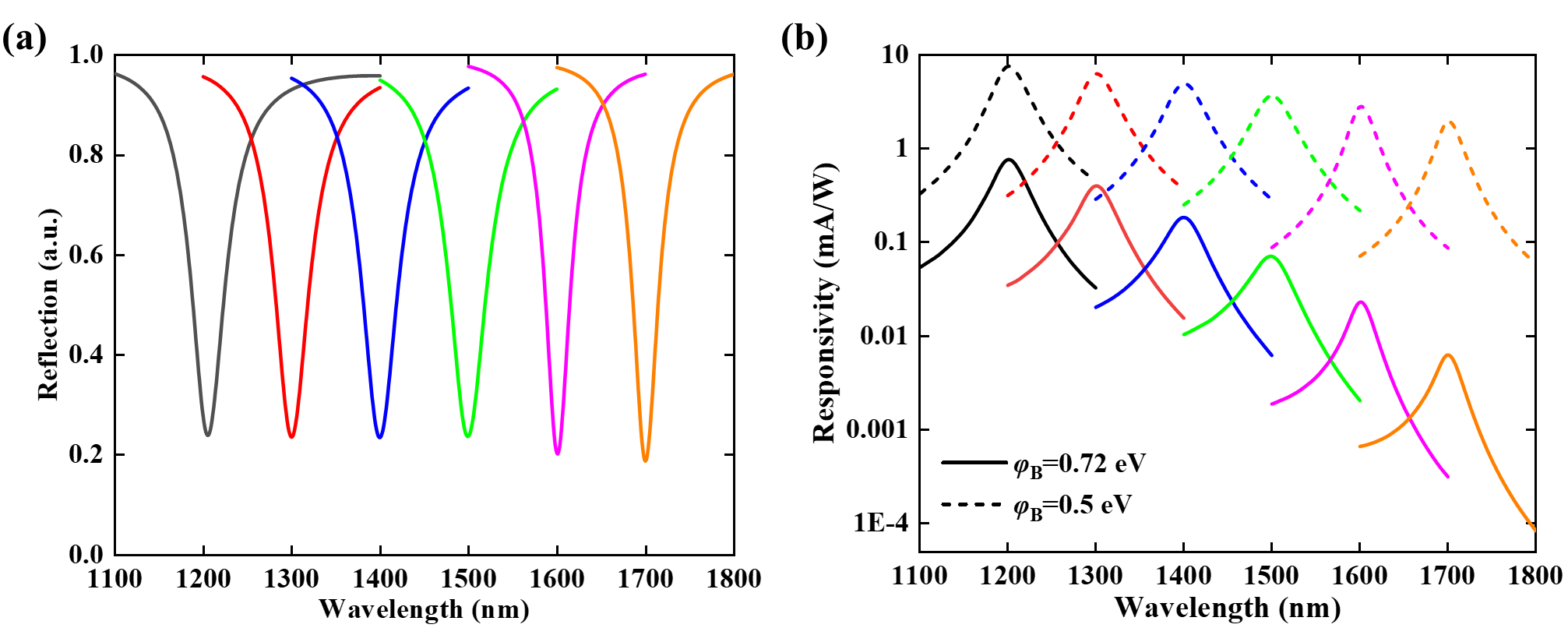


**Supplementary Figure S3.** Designed six devices at different resonant wavelengths. (a) The simulated reflectance spectra and (b) calculated responsivities of these devices with *φ*_B_ = 0.72 eV (solid lines) and *φ*_B_ = 0.5 eV (dash lines).

**Supplementary Table S1.** Core parameters and performances of TP-based HE NIR photodetectors based on other structural principles include simulation results and experimental results.

| **Device configuration** | | $\boldsymbol{\lambda}_{\boldsymbol{DBR}}$  **(nm)** | **FWHM (nm)** | **Peak photoresponsivity**  **(mA/W)** | **Ref** |
| --- | --- | --- | --- | --- | --- |
| **Simulation results** | Silica substrate/Au/ZnO/Au/(TiO_2_/Al_2_O_3_)^8^ | 813 | 8 | 0.0137 | [31] |
|  | (Silica substrate/Si)^6^ /(TiO_2_/Au)^4^ | 1140 | 50~100 | 0.4 | [28] |
|  | Silica substrate/(TiO_2_/SiO_2_)^9^/Au | 892 | 14 | 0.75 | [34] |
|  | Si/(TiO_2_/Al_2_O_3_)^7^/Si/TiN | 1140 | 239.3 | 26.1 | [32] |
|  | 1DPC/Au/TiO_2_/1DPC | 800 | 0.4 | 21.87 | [38] |
|  | Au/Si/Au/(TiO_2_/SiO_2_)^6^ | 1100 | 20 | 16 | [35] |
|  | Silica substrate /Au/(MoS_2_/SiO_2_)^4^ | 988 | 12 | 12.1 | [39] |
|  | Silica Substrate/Au/(Si/SiO_2_)^7^ | 1550 | 6 | / | [40] |
| **Experimental results** | Silica/(Ge/SiO_2_)^3^/Ge/SiO_2_/ITO/ZnO/Ti/Au | 1581 | 43 | 0.00826 | [33] |
|  | Au/Ti/Si substrate/Au/(TiO_2_/SiO_2_)^5^ | 1450  1400  1580 | 52  32 | 0.11  0.195  0.012 | This work |

**Supplementary Table S1. TP-based hot-electron photodetector in literatures**

For better comparison, the key parameters of excellent TP-based hot-electron photodetectors in recent years are given in Table S1, listing their central wavelength, FWHM, and Peak photoresponsivity. It is worth noting that most of the currently reported TP-based detectors are mainly based on simulation results, with fewer experimental reports. Moreover, a Si-based hot electron NIR photodetector based on TPs has not been reported. We proposed a silicon-based narrowband planar hot-electron photodetector with Tamm plasmons, the responsivity of the device reaches 0.195 mA/W at a wavelength of 1400 nm.

22. Dong, Y.; Li, J.; Liang, W.; Nan, X.; Wen, L.; Chen, Q. CMOS-Compatible Broad-Band Hot Carrier Photodetection with Cu–Silicon Nanojunctions. *ACS Photonics* **2022**, 9 (11), 3705-3711

28. Shao, W. J.; Yang, Q. R.; Zhang, C.; Wu, S. L.; Li, X. F. Planar dual-cavity hot-electron photodetectors. *Nanoscale* **2019**, 11 (3), 1396-1402.

31. Zhang, C.; Wu, K.; Giannini, V.; Li, X. F. Planar Hot-Electron Photodetection with Tamm Plasmons. *ACS Nano* **2017**, 11 (2), 1719-1727.

32. Wang, J. Y.; Zhu, Y. S.; Wang, W. H.; Li, Y. Z.; Gao, R.; Yu, P.; Xu, H. X.; Wang, Z. M. Broadband Tamm plasmon-enhanced planar hot-electron photodetector. *Nanoscale* **2020**, 12 (47).

33. Wang, Z. Y.; Clark, J. K.; Ho, Y. L.; Delaunay, J. J. Hot-electron photodetector with wavelength selectivity in near-infrared via Tamm plasmon. *Nanoscale* **2019**, 11 (37), 17407-17414.

35. Li, R. F.; Zhang, C.; Li, X. F. Schottky hot-electron photodetector by cavity-enhanced optical Tamm resonance. *Appl Phys Lett* **2017**, 110 (1), 5.

38. Liang, W. Y.; Xiao, Z.; Xu, H. T.; Deng, H. D.; Li, H.; Chen, W. J.; Liu, Z. S.; Long, Y. B. Ultranarrow-bandwidth planar hot electron photodetector based on coupled dual Tamm plasmons. *Opt. Express* **2020**, 28 (21), 31330-31344.

39. Zhu, Y. S.; Yu, P.; Liu, T. J.; Xu, H. X.; Govorov, A. O.; Wang, Z. M. Nanolayered Tamm Plasmon-Based Multicolor Hot Electron Photodetection for O- and C-Band Telecommunication. *Acs Appl Electron* Ma **2021**, 3 (2), 639-650.

40. Zhou, C. F.; Wang, Z. Y.; Ho, Y. L.; Shiomi, J.; Delaunay, J. J. Optimized Tamm-plasmon structure by Differential Evolution algorithm for single and dual peaks hot-electron photodetection. *Opt Mater* **2021**, 113.
